# Surface Chemistry and Particle Morphology Changes in Pine Biomass under Indirect Thermal Gradients: Implications for Feed Screw Design

**DOI:** 10.1021/acsomega.5c08658

**Published:** 2025-12-02

**Authors:** Yining Zeng, Josephine Gruber, Tim Dunning, Steven Rowland, Daniel Carpenter, Bryon S. Donohoe

**Affiliations:** † Renewable Resources and Enabling Sciences Center, 53405 National Renewable Energy Laboratory Golden, Golden, Colorado 80401, United States; ‡ Biosciences Center, National Renewable Energy Laboratory, Golden, Colorado 80401, United States

## Abstract

The conversion of biomass feedstocks into fuels and chemicals using fast pyrolysis is a promising approach to renewable energy. Feed screws that convey biomass to pyrolysis reactors, however, often encounter plugging. Indirect heating of the feed screw occurs due to contact with the pyrolysis chamber, resulting in a heating gradient ranging from ambient temperature (22 °C) to reactor temperature (500 °C). Given that major cell wall macromolecules, such as lignin, cellulose, and hemicellulose, begin to produce bio-oils and volatile resin acid compounds within this temperature gradient, we hypothesized that indirect heating during feed screw conveyance is sufficient to cause premature degradation of biomass. We characterized this degradation by observing increases in surface roughness, changes in overall particle morphology, and the production and deposition of bio-oils on biomass particle surfaces. Correlative analysis between optical in situ hot-stage microscopy, confocal Raman spectroscopy, and SEM analysis revealed that heating at temperatures as low as 375 °C caused significant increases in surface roughness, with large fissures forming between and within cell walls. Additionally, droplets of bio-oil were observed on particles, especially in the bark and cambium samples. This work suggests that these phenomena contribute to particle agglomeration, leading to feed screw plugging, and that engineering a solution to cool the feed screw could prevent particle agglomeration and reduce plugging incidents, thereby increasing biomass processing efficiency.

## Graphical abstract

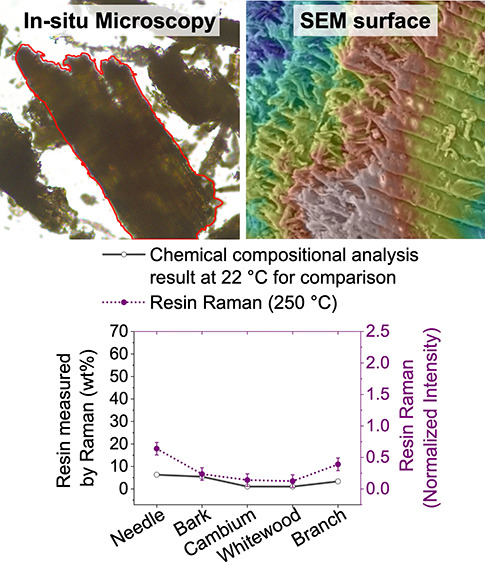

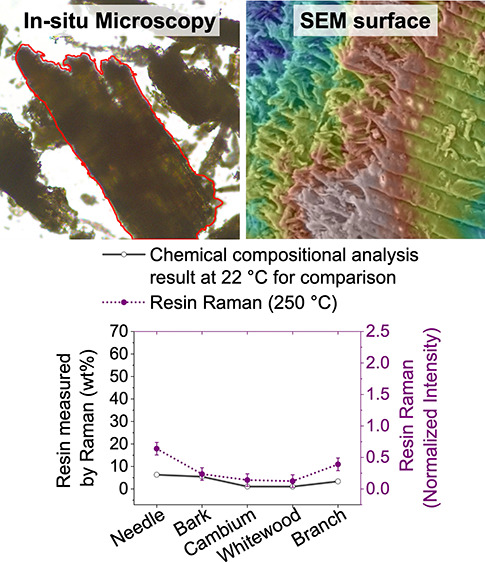

## Introduction

Increasing interest in bolstering domestic supply chains and revitalizing rural economies has spurred research into renewable feedstocks for fuels and chemicals.^1–3^ Lignocellulosic biomass—primarily waste from food crop production, timber harvest, and forestry residues such as pine—offers a plentiful and underutilized resource for value-added conversion.^4–6^ This biomass can be processed through chemical, biological, thermal, and mechanical methods to yield environmentally friendly fuels, including combustible gases, biodiesel, and bio-oil. Among these approaches, fast pyrolysis in a fluidized bed reactor is widely regarded as one of the most efficient thermal conversion methods.^6–9^ However, industrial implementation can be hindered by challenges such as feed screw plugging, which is often caused by the indirect heating of the feedstock as it transitions from ambient temperatures (∼22 °C) to reactor operating temperatures (∼500 °C). Targeted conversion pathways also enable the production of specialty chemicals and food additives, enhancing both the sustainability and profitability of biomass utilization.^4,5^

Pine forestry residue, a source of lignocellulosic biomass, comprises materials left at cut sites, including treetops, needles, bark, and branches, creating a highly variable biomass feedstock with a wide range of plant tissue types, volatiles, and viscous substances that can be exuded during processing or pyrolytic heating.^1,10^ Current literature suggests that fast pyrolysis is the most effective method for overcoming this variability and efficiently converting forestry residue to biofuels. Characterized by rapid heating (less than 1 second) to temperatures between 400 and 700 °C, fast pyrolysis shows promise for generating bio-oil and gas that could serve as fuel alternatives.^4,5^ In fluidized bed pyrolysis reactors, preheated sand is fluidized with a continuous flow of hot gas, often nitrogen, to rapidly heat biomass particles.^4^ However, feed screws that move biomass into reactors frequently encounter the problem of plugging, decreasing operation times and hampering biomass conversion productivity. Feed screws are heated indirectly due to their proximity to the heated pyrolysis reactor, experiencing temperatures anywhere between ambient temperature (22 °C) and the temperature of the reactor (500 °C).^5,7^ The impact of this heating regime on pine forestry residue particles conveyed to the reactor is poorly understood, as is the mechanism behind the plugging phenomenon.

Premature heating of biomass during feed screw conveyance can significantly alter particle morphology, affecting how particles flow and potentially leading to plugging. Experimental studies show that heating biomass to 500 °C induces marked morphological transformations. Below 300 °C, cell walls and lumens expand and swell as hemicellulose and lignin begin to degrade, releasing gases such as hydrogen, hydrocarbons, and water vapor.^6^ As temperatures exceed 350 °C, degradation of cell wall macromolecules, internal gas pressure, and carbonization drive the collapse of the cell wall and lumen.^11^ Similar structural breakdowns have been observed in diverse biomass types, including Brazil nut shells and hardwoods, where heating between 350 and 500 °C results in ruptured cell walls, lumen deconstruction, and particle shrinkage.^11^ Surface texture changes—such as large pore formation and wrinkling caused by volatilization and structural collapse—increase particle surface area and interparticle friction. Notably, at temperatures as low as 200 °C, the breakdown of cell wall components can also generate highly viscous bio-oils, which may coat particle surfaces and promote agglomeration.^12–14^ Given that feed screw environments may expose biomass to temperatures within this critical range, we hypothesize that resulting changes in particle morphology and surface texture, along with bio-oil formation, contribute to increased interparticle friction and agglomeration, ultimately leading to feed screw plugging.^15,16^

Temperature has also been known to affect surface texture, namely porosity and surface area, of biomass in ways that could increase interparticle friction and cause plugging of the feed screw. Pyrolysis of nutshells and poplar wood indicates that the volatilization of cell wall macromolecules and intracellular components releases gases and liquids that create pores in the particle surface.^6,11^ As the heating temperature increases, pores expand, eventually joining to form larger pores. As a result, the cell wall breaks down further, causing surface wrinkling that, in addition to the increase in porosity, increases particle surface area and dramatically alters the surface texture.^6,11^ Contrary to these observations, loblolly pine chips subjected to various pyrolysis temperature regimes exhibited maximal porosity and surface wrinkling from 300 to 350 °C. Surface pores contracted at higher temperatures (over 700 °C), a phenomenon attributed to the restacking of graphitized carbon.^17^ So, while the temperature regime within the feed screw is expected to be sufficient to cause significant changes in particle surface texture, the exact nature of this change is unclear.

Pine biomass pyrolysis is conducted primarily to produce bio-oils, although premature bio-oil production can have detrimental effects on the pyrolysis machinery. Bio-oil is a highly viscous, heterogeneous mix of water, lipids, and solids.^5,18,19^ In addition, the mixture of polar and nonpolar liquids leads to the formation of micelles, which can cause significant particle agglomeration within the reactor or associated machinery.^20^ Also corrosive, bio-oil can damage feed screws, further exacerbating particle agglomeration.^18,21^ Therefore, the anatomical fraction and its relative composition of cellulose, hemicellulose, and lignin could begin releasing bio-oil components during feed screw conveyance of pine forestry residue. Hemicellulose begins to break down at temperatures as low as 200 °C, and lignin can start to break down near 280 °C. Particles within the feed screw experience temperatures this high and higher, indicating premature bio-oil production is likely.^10,22^ With as much as 50% optimal yield of bio-oil being produced by heating to temperatures as low as 350 °C, premature bio-oil production during feed screw conveyance is a concern despite the short residence time in the feed screw.^3,21^

Previous work characterizing the effects of pyrolysis on pine anatomical fractions’ low-temperature pyrolysis of similar biomass suggests that the temperature regime particles experience during feed screw conveyance may contribute significantly to particle agglomeration that causes feed screw plugging. We hypothesize that particles of different anatomical fractions (bark, cambium, needles, and whitewood) will undergo dramatic physical and chemical changes during this heating regime, increasing particle friction and cohesion, and contributing to feed-screw plugging. In this work, we introduce a correlative, particle-matched workflow that maps how indirect feeder-style heating (22–500 °C) drives temperature-specific morphology and surface-chemistry changes in pine residues and ties those changes mechanistically to plugging risk. The objective of this study was to determine how indirect thermal exposure in a screw feeder (22–500 °C) alters the morphology, surface texture, and near-surface chemistry of pine forestry-residue particles and how these changes contribute to interparticle friction, agglomeration, and feed-screw plugging during fast pyrolysis.

## Methods

**Pine forestry residue material** was sourced from loblolly pine trees (*Pinus taeda*) harvested from a plantation in Edgefield, South Carolina. Harvesting conditions were dry, with less than 1/2 in. of rainfall received within 10 days prior to harvest. Whole trees were cut at the stump approximately 4 in. above ground, topped, and limbed, and 4 in. sections were cut from the bole using a chainsaw. The forestry residues were segregated into needles, branches, bark, whitewood, and cambium fraction anatomical fractions at Idaho National Laboratory. The resulting material was milled to 2 mm via a Wiley knife mill (Thomas Wiley Model 4) with a 0.5 mm material retention sieve and stored at room temperature in airtight containers.

**Optical in-situ hot stage microscopy** was used to monitor relative changes in particle size during heating. Observations were conducted using a Nikon E800 confocal microscope (Nikon, Japan) with a 10x long working distance objective. Particles of a single anatomical fraction were arranged between glass coverslips and heated from 22 to 500 °C at a rate of 5 °C/min with a Linkam T95 (Linkam Scientific Instruments, Epsom, United Kingdom) hot stage, kept under approximately 2 psi vacuum and 2 psi nitrogen gas. Five replicates of each anatomical fraction (needles, branches, bark, whitewood, and cambium) were heated and imaged every 30 s using a SPOT Optics RTKE 5.1 camera (Diagnostic Instruments, Sterling Heights, MI). Image processing using FIJI^23^ included aligning images with linear stack alignment in the SIFT plugin,^24,25^ converting to binary using an IsoData threshold, and measuring particle area using the Analyze Particles function. Changes in particle surface area relative to their starting surface area were modeled in R^26^ as a function of time using a generalized additive model (GAM) and a 95% continuous confidence interval.^27^ This model identified 250 and 375 °C as temperatures of significant morphological change, relevant to further characterization via confocal Raman spectroscopy and scanning electron microscopy (SEM) and to identify changes in particle morphology, surface texture, and any compounds that may emerge on particle surfaces during heating.

To prepare samples for **confocal Raman spectroscopy and SEM**, particles of a single anatomical fraction were arranged between glass coverslips, heated to 250 °C, and imaged every 30 s using the system described above. Upon reaching the target temperature, the sample was quenched with liquid nitrogen using a Linkam LNP95 liquid nitrogen cooling system operating at a maximum cooling rate and returned to room temperature (under 90 s on average). This protocol was repeated to prepare ten samples of each anatomical fraction, five heated to 250 °C and five heated to 375 °C. Samples were retained in glass coverslips for further analysis.

Heated samples were analyzed by confocal Raman spectroscopy to determine the chemical identity of volatilized compounds that emerge on particle surfaces and to identify the macromolecular degradation sites. Samples were photobleached to reduce autofluorescence before confocal Raman analysis. Signal integration times were optimized to obtain a minimum of 80% intensity and subjected to 30 accumulations for optimal SNR. For each sample, four Raman spectra were collected on a Horiba LabRAM HR confocal Raman. When possible, ROIs identified during optical microscopy were analyzed for correlative analysis. Major functional peaks and likely chemical compounds identified by mass spectrometry of heated pine forestry residue were placed in the averaged Raman spectra and compared between heating regimes across all anatomical fractions.

Samples analyzed by confocal Raman spectroscopy were then transferred to carbon tape and coated with 12 nm of iridium for scanning electron microscopy (SEM). SEM micrographs were collected at 20 kV by using an FEI Quanta 400 FEG instrument under low vacuum, operating with the gaseous solid-state detector (GAD) to capture secondary electrons. ROIs identified via optical microscopy and confocal Raman spectroscopy were imaged by correlative imaging to analyze the particle morphology and surface texture when possible. Surface texture analysis was performed on SEM micrographs using the Surf 3D reconstruction plugin developed in ImageJ software.^28^ To further quantify changes in the particle surface texture, we collected tilt pairs of SEM micrographs angled 15° apart. These tilt pairs were analyzed using the Mountains software package (www.digitalsurf.com) to generate height maps.^29^ The surface texture was compared among samples subjected to the 250 and 375 °C heating regimes across anatomical fractions. The particle surface area was measured per frame to determine temperature regions with significant morphological changes. Samples were heated to 250 and 375 °C and analyzed by confocal Raman spectroscopy and SEM.

In summary, we heated fractionated pine residues from ∼22 to 500 °C in a hot-stage microscope, used image analysis to identify transformation inflections, and quenched samples at 250 and 375 °C for correlative analysis. Samples from these conditions were characterized by confocal Raman spectroscopy, SEM with 3D stereometry, and image-based morphometry to link surface chemistry and texture changes to particle shrinkage and swelling behavior relevant to feed-screw plugging.

## Results and Discussion

To evaluate the separation and variability of the anatomical fraction samples, we observed the particles by stereomicroscopy. Figure 1 shows stereomicrographs of pine residue anatomical fractions milled to 2 mm or smaller.

**Figure 1:**
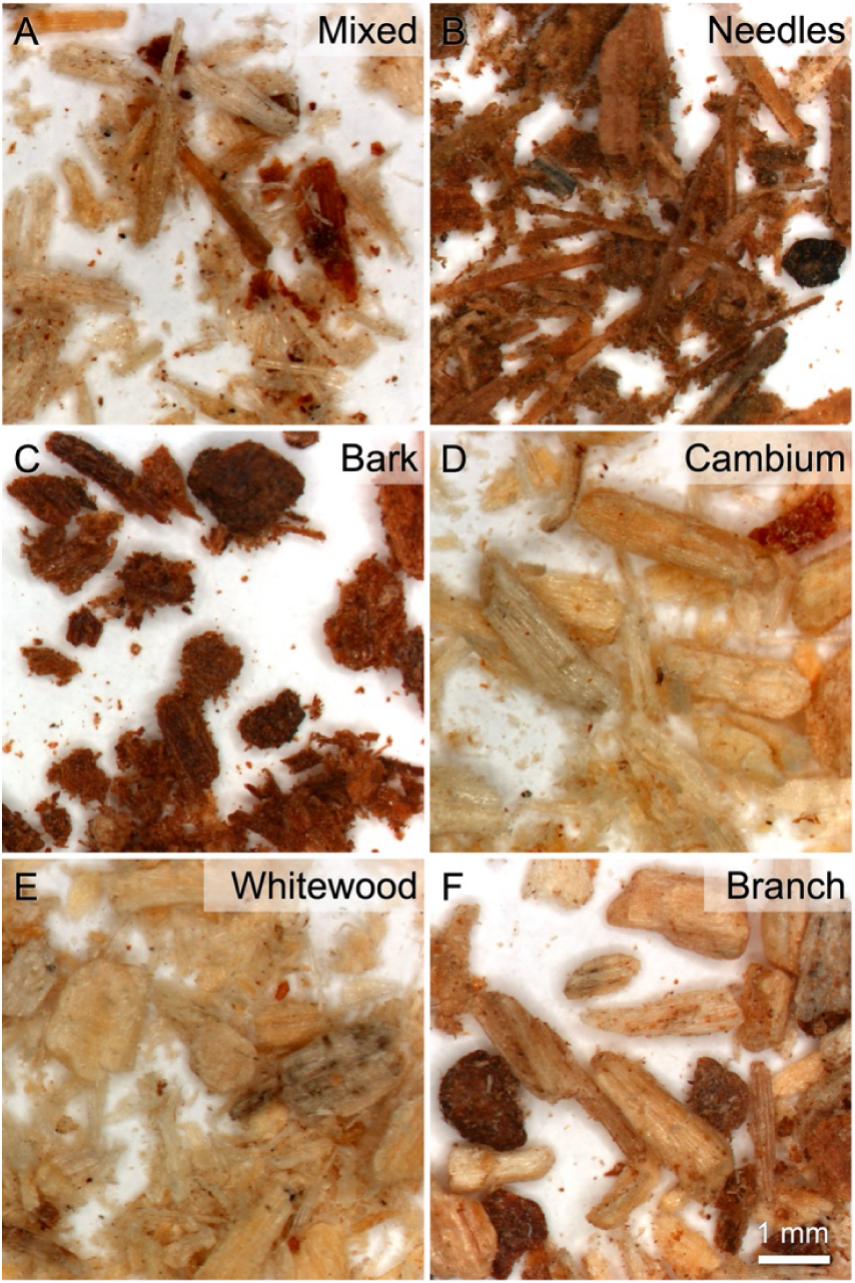
Pine residue anatomical fractions milled to 2 mm or less are shown in stereo micrographs. The mixed sample (A) contains all five of the other fractions, including needles (B), bark (C), cambium (D), whitewood (E), and branch (F). The branch sample (F) by its nature also includes all four of the distinct tissue types (needles, bark, cambium, and whitewood). Scale = 1 mm.

The mixed sample includes all of the distinct tissue fractions including needles, bark, cambium, and whitewood. This diversity in tissue types suggests inherent variability in the physical and chemical attributes of the feedstock and behavior during subsequent thermal processing. The needle fractions contained mostly needles, with some bark contamination. The bark sample appeared to be almost entirely bark tissue. The cambium and whitewood samples looked similar, and we identified some whitewood in the cambium sample. As expected, the branch sample contains a mixture of needle, bark, cambium, and whitewood tissue particles. Based on these observations, we expected that the cambium and whitewood may behave similarly and that the branch samples would perform as an average of the other tissue types.

### Hot Stage Microscopy to Reproduce Screw-Feeder Temperature Profiles

To mimic the thermal environment experienced by the pine residues in the feeder system of the National Renewable Energy Laboratory’s (NREL) pyrolysis reactor, we placed the various fractions in an in situ heating stage connected to a microscopy platform. This setup enabled us to control the temperature range from ambient (22 °C) to the temperature at the end of the feed system as it enters the pyrolysis reactor (500 °C).^30^ We sandwiched a few pine residue feedstock particles at a time between two coverslips, heated the system to 500 °C, and captured bright-field transmitted light images every 30 s to monitor changes in particle morphology.

In Figure 2, optical bright-field micrographs illustrate particle morphology transformations under this thermal treatment, captured at ambient (22 °C), 250 °C, and 375 °C.

**Figure 2:**
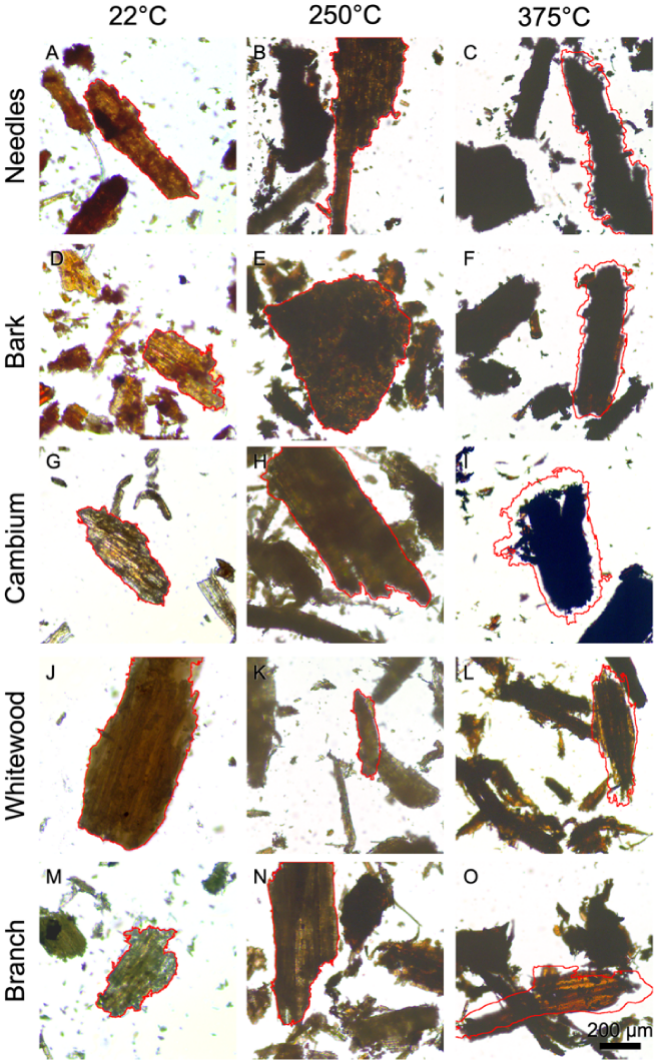
Optical bright-field micrographs depicting pine residue particles subjected to heating at temperatures of 22 °C (A, D, G, J, and M), 250 °C (B, E, H, K, and N), and 375 °C (C, F, I, L, and O). The images were obtained during a continuous heating experiment on an in situ microscope stage. Each panel is a frame from a separate in situ experiment to show some of the measured particle variability. The red outlines represent the particle size and shape before heating, facilitating particle shrinkage visualization. Scale bar = 200 μm.

Images collected during continuous in situ heating experiments document progressive particle shrinkage after a slight swelling at around 250 °C. The red outline annotations mark the original size of the selected particle at 22 °C and highlight the marked reduction in particle dimensions by 375 °C. The images show that each of the tissue types remains unchanged in size or increases slightly by 250 °C, but all have shrunk substantially by 375 °C. The cambium and whitewood fractions display particularly dramatic volume reductions, indicating substantial thermal degradation.

To quantify the changes in particle size, we applied a generalized additive model to plot the areas measured by segmenting the particles in the images captured from the in-situ hot stage experiments and measuring their area. Figure 3 shows the results of using generalized additive modeling (GAM) to quantitatively describe relative particle size changes across anatomical fractions during thermal treatment from 22 to 500 °C.

**Figure 3:**
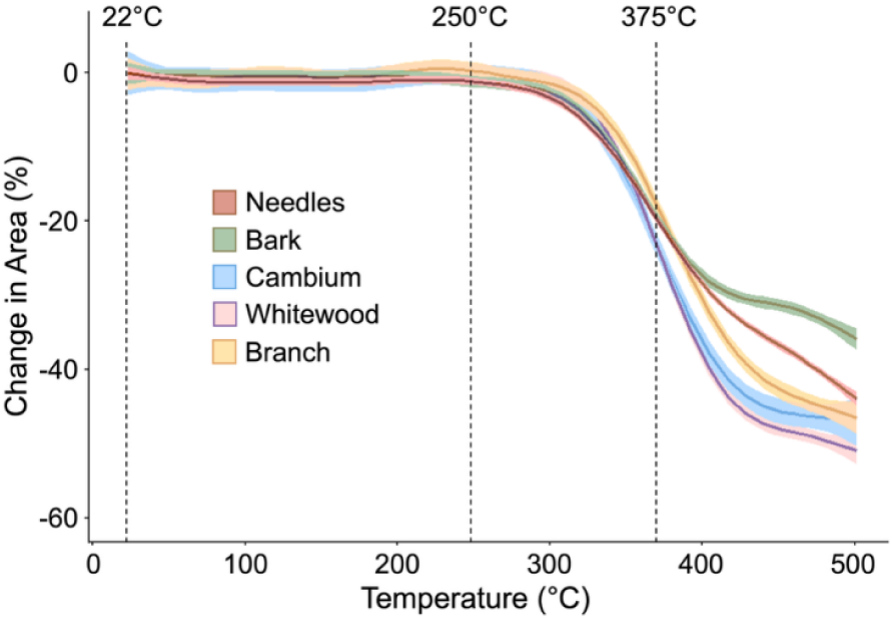
Generalized additive model (GAM) plots illustrate relative particle size changes among anatomical fractions (bark, cambium, needles, whitewood, and branches) derived from optical in situ micrographs obtained during heating. The shaded areas of the model represent a 95% confidence interval. The particle heating replicated screw feeder conditions, transitioning from ambient temperature (22 °C) to pyrolysis reactor temperature (500 °C) at a heating rate of 5 °C/min.

The GAM plots underscore critical morphological transitions, particularly between 300 and 400 °C, with the rate of particle shrinkage peaking around 375 °C. Slight particle swelling around 250 °C is observed in the cambium and branch fractions, suggesting some early-stage thermal softening and gas evolution.

This image analysis revealed that the most significant changes in particle size occurred between 300 and 400 °C, with the maximum rate of change near 375 °C. The branch and cambium samples showed evidence of slight swelling near 250 °C. We chose to focus on both temperature regions to further characterize the heated residue samples by scanning electron microscopy (SEM) and Raman spectroscopy to investigate potential changes in surface texture and surface chemistry.

### Particle Morphology and Surface Texture Analysis Reveal the Formation of Fissures and Droplets

To investigate changes in the surface texture and topography that may take place during this heating profile, we analyzed the biomass particle surfaces by scanning electron microscopy (SEM). Images of representative surfaces from each anatomical fraction are shown in Figure 4.

**Figure 4:**
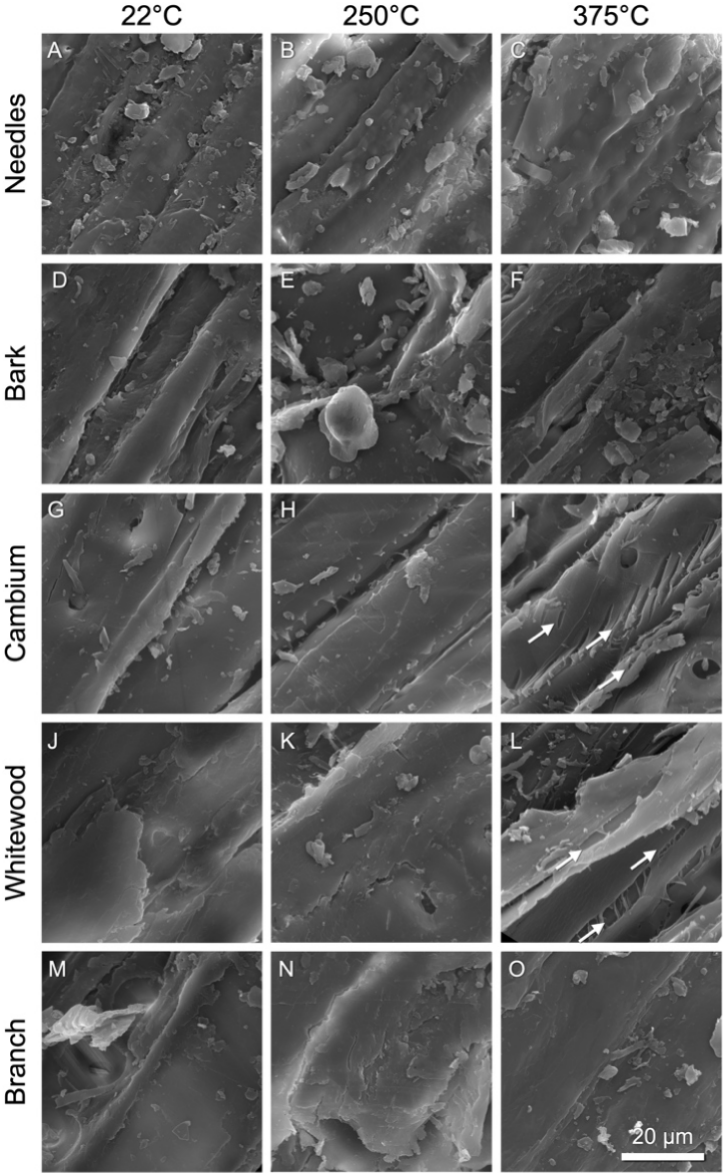
SEM micrographs of specific regions of the needle (A–C), bark (D–F), cambium (G–I), whitewood (J–L), and branch (M–O) particles that underwent optical in situ heating experiments, as illustrated in Figure 2. The analysis of surface topology identifies instances of corrugated surfaces featuring fissures that develop between cells and within cell walls, which enhance the overall roughness. Fissures (white arrows) are most prominent in the cambium (I) and whitewood (L) samples at 375 °C. Scale bar = 20 μm.

Figure 4 utilizes scanning electron microscopy (SEM) to provide high-resolution views of the particle surface morphological changes following heating. Detailed examination reveals significant surface alterations characterized by fissure formation between and within cells, notably at 375 °C. Cambium and whitewood fractions show prominent fissuring (white arrows), suggesting severe structural compromise, likely affecting interparticle friction and mechanical stability.

Qualitative inspection of the surfaces revealed a wide range of variability among the particle surfaces. One consistent phenomenon was the appearance of multiple cracks on the surfaces of particles around 375 °C. In particular, the cambium and whitewood samples displayed these surface cracks at high temperatures (Figure 4I and 4L, arrows).

To quantify changes in the particle surface texture, we collected tilt pairs of SEM micrographs angled at 15°. These tilt pairs were analyzed using the Mountains software package (www.digitalsurf.com) to create accurate height maps. Figure 5 presents stereometric 3D reconstructions derived from SEM micrographs, depicting variations in surface roughness at ambient conditions (22 °C) and following heating to 250 and 375 °C.

**Figure 5:**
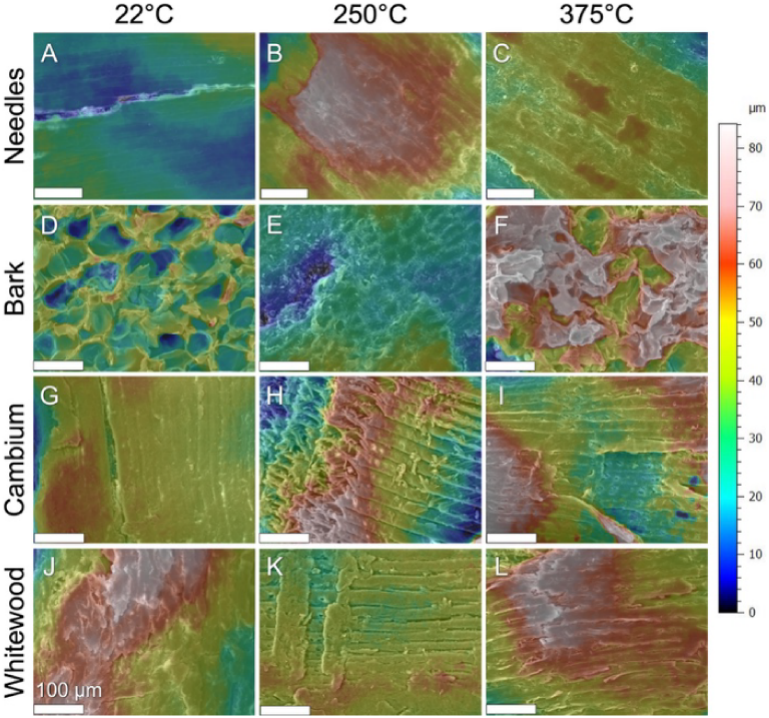
3D stereometric reconstructions of selected SEM micrographs, illustrating the surface topology of needle, bark, cambium, and whitewood tissues at ambient temperature (22 °C) (A–J), as well as heated at temperatures of 250 °C (B–K) and 375 °C (C, F, I, L). Qualitative analysis indicated that needles (A–C) exhibited increased surface roughness at 250 °C, whereas bark (D–F), cambium (G–I), and whitewood (J–L) demonstrated some increase in roughness at 375 °C. Values exceeding 80 μm are represented in white. Scale bars = 100 μm.

Needle surfaces become noticeably rougher at 250 °C, while bark remains unchanged across all temperatures. Cambium and whitewood exhibit marked increases in surface roughness at 375 °C, correlating with pronounced fissure development and particle shrinkage. Figure 6 provides quantitative surface roughness analysis via box plots of root-mean-square height (Rq) values obtained from stereometric reconstructions. The data confirm that needle and cambium fractions exhibit increased roughness starting at 250 °C. At 375 °C, whitewood substantially increases surface roughness, corroborating visual observations from SEM images. Conversely, bark tissue maintains a stable surface texture throughout the thermal range examined.

**Figure 6:**
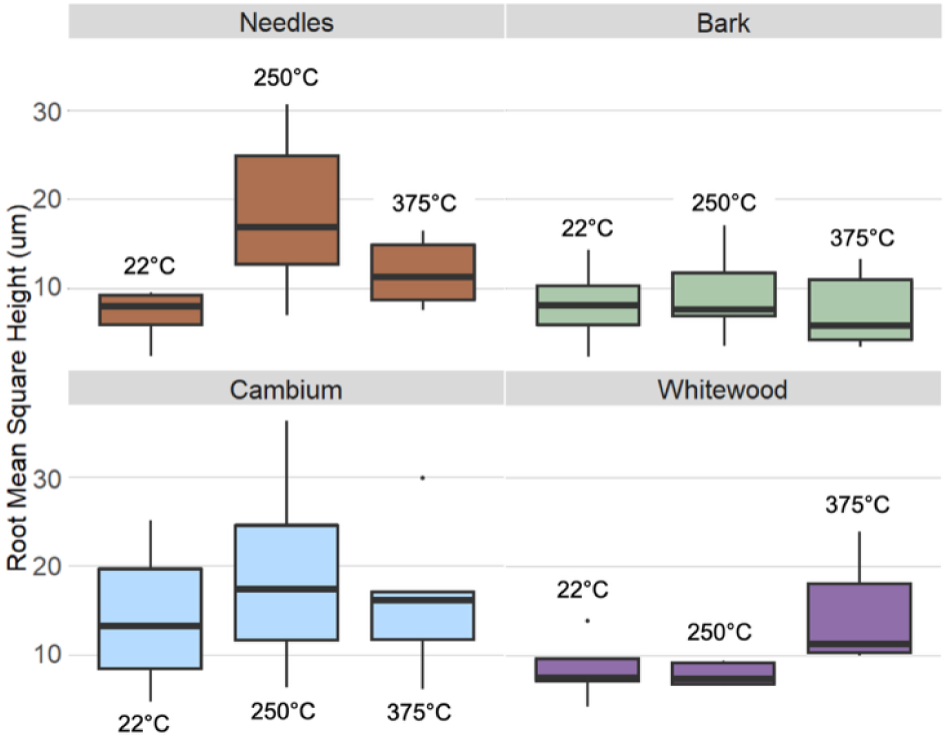
Surface roughness profiles derived from SEM stereometry. Boxplots of Rq (root-mean-square height) values obtained from stereometric 3D reconstructions at 22 °C (left), 250 °C (middle), and 375 °C (right) indicate that needles (brown) and cambium (blue) increase in roughness at 250 °C. In contrast, whitewood (purple) surface roughness increases at 375 °C. Bark (green) exhibits no appreciable change in roughness due to heating.

Surface roughness parameters include root-mean-square height (Rq) (Figure 6), maximum peak height (Figure S1), and maximum valley depth (Figure S1).

This quantitation confirmed significant variability in particle surface texture across all anatomical fractions and at all temperatures. Minor changes in surface texture were observed in particles heated to 250 °C, with cracks separating cells and fissures within cell walls becoming more evident. At 375 °C, particle surfaces become dramatically rougher, with large cracks in and between the cell walls predominating. One notable pattern is that the surface texture of the bark material does not change at any temperature by any measure (Figures 6 and S1). Another pattern that emerged is that whitewood showed the greatest change of any tissue type, with markedly increased surface roughness at 375 °C.

### Formation of Surface Droplets and Chemical Degradation Accompany Morphological Changes

We were initially interested in potential changes in surface properties from a mechanical perspective. Increasing surface roughness could impact particle interactions in a way that promotes clogging. However, as we zoomed into the particle surfaces, we observed evidence of another phenomenon. Small (<200 μm) droplets appeared on the surfaces of the particles from all the tissue types at 375 °C (Figure 7).

**Figure 7:**
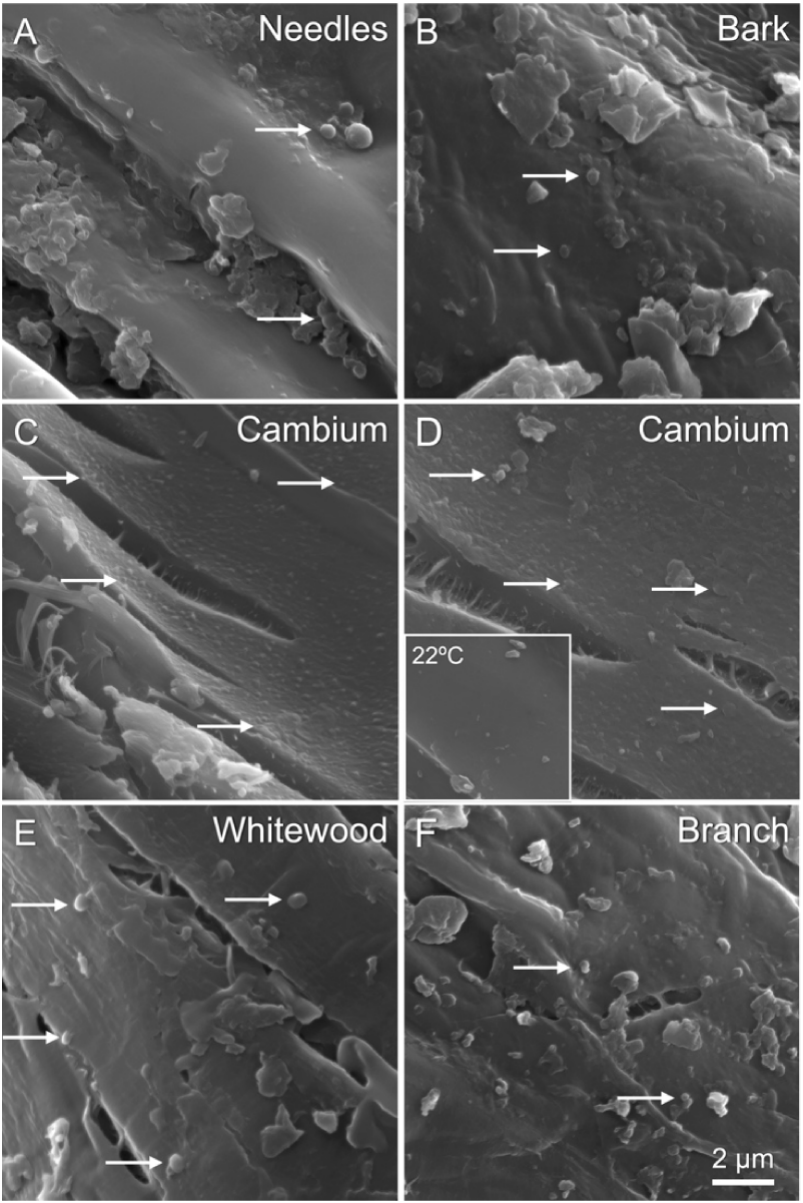
Droplets of redeposited or migrated biomass compounds adhered to the surface of particles were observed in all samples heated at 375 °C and then quenched (A, F). The surface of the cambium samples was uniquely coated with small (less than 500 nm) droplets (C, D). Top (left to right): abundant droplets, especially near fractured surfaces. Scale bar = 2 μm.

In Figure 7, SEM analysis reveals the appearance of small droplets (<500 nm) adhering to the surfaces of particles that had been heated to 375 °C, indicating redeposition or migration of volatile biomass-derived compounds. These droplets are notably abundant near fissure sites, particularly on cambium particles, suggesting localized thermal degradation and bio-oil formation that may enhance particle agglomeration and plugging phenomena within pyrolysis systems.

In most of the tissue types (needles, bark, whitewood, and branches), those droplets appear as sparse, semispherical droplets (Figure 7A, B, E, F, arrows), with the whitewood displaying slightly more than the other tissues (Figure 7E, arrows). On the other hand, the cambium tissue appeared largely coated with droplets (Figure 7C, D, arrows). The inset image in Figure 7D is a reminder of how relatively smooth and clean the surface of the cambium particles can appear at ambient temperature. Interestingly, observed bio-oils were commonly located near fissures in the cell wall caused by heating. Increased surface roughness and possible evolution of bio-oils caused by premature heating during feed screw conveyance may increase interparticle friction and cohesion. We performed Raman spectroscopy on the surfaces of the heated particles to learn more about the composition of these droplets.

### Surface-Oriented Composition Estimation Integrating the Raman Signal Correlates with Bulk Measurements and Detects Resin Aggregates

Figure 8 presents Raman spectroscopy data for anatomical fractions previously characterized microscopically.

**Figure 8:**
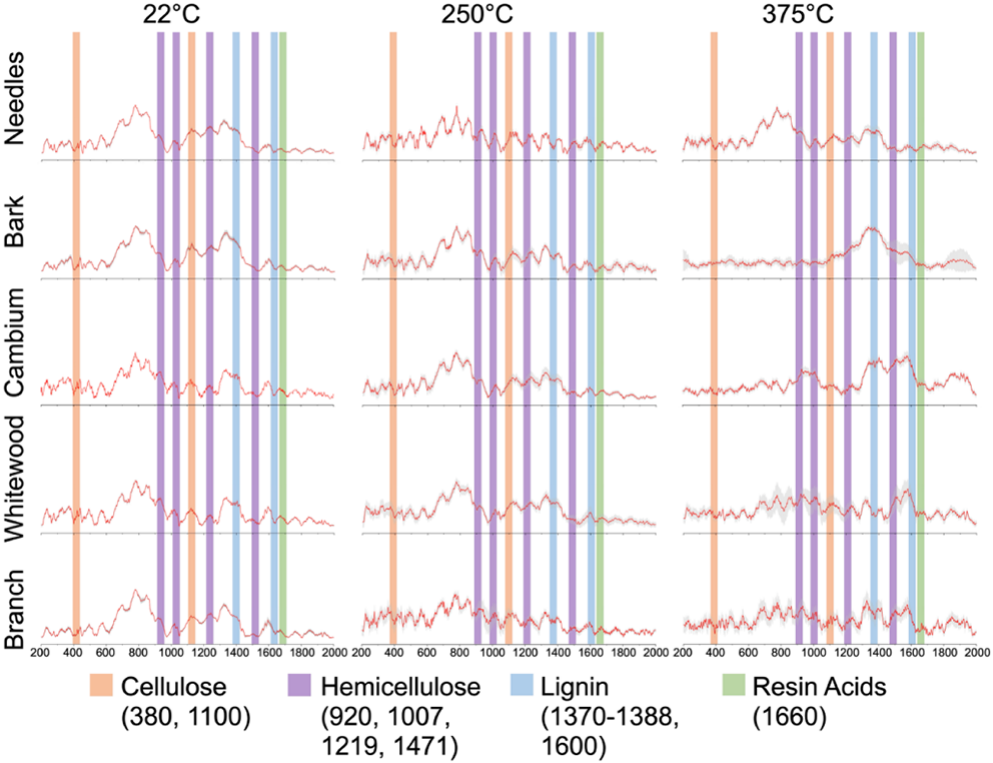
Raman spectra of the samples previously examined by scanning electron microscopy (SEM). Each spectrum represents the average of three replicate measurements per sample, with shading denoting the standard error. Bands associated with cell wall macromolecules (cellulose—orange, hemicellulose—purple, and lignin—blue) and volatile resin acids (green) were identified.

Averaged spectra illustrate distinct chemical signatures corresponding to cellulose, hemicellulose, lignin, and volatile resin acids. Notably, heating induces discernible decreases in cellulose and hemicellulose signals, reflecting progressive degradation of these structural macromolecules and consistent with observed morphological transformations. All anatomical fractions show decreased signals in cellulose regions (orange), suggesting cell wall degradation is occurring. Cambium samples also showed reduced lignin signals (blue), and needle samples show decreased resin acid signal in the 1660 cm^–1^ region, although the signal is highly variable between replicates.

The Raman signal from biomass was weak. To improve the signal-to-noise ratio, long integration times were used to maximize the Raman signal. The integration time was chosen so that the total signal, including the fluorescence background, was approximately 80% of the full dynamic range of the detector. For the same spectrum, many repetitions were used to improve the signal-to-noise ratio. The typical total acquisition time for a spectrum was ∼15 min. Then, the fluorescence background was subtracted. To cover the possible variations of the Raman bands from sample particle to particle, many particles from the batch of the sample were measured by using the same procedure. To pool the results from many particles together, each spectrum (after individual fluorescence background subtraction) was normalized to avoid differences in the mean intensity between spectra. The average spectrum and the standard error at each Raman wavelength were determined. The shading in Figure 8 shows the standard error.

To make a valid comparison between heat treatments or biomass types, the Raman intensity across a 2 cm^–1^ band for the signature of interest was integrated to avoid possible uncertainty introduced by using a single data point in the spectrum. The Raman signature bands for cellulose, hemicellulose, and lignin were selected according to our previous assignments.^31^ The resin Raman signature bands were selected according to previously published results. Table S1 shows the Raman assignments and the locations where the background baseline was chosen. A local background was determined, and a background subtraction was performed to obtain the integrated peak intensity (Supporting Information, Table S1).

To include as much Raman information for a species as possible, the total Raman signal for each species was calculated by summing up the results from all of the possible Raman signature bands of this species. For example, for cellulose, both 380 and 1100 cm^–1^ were selected. A 2 cm^–1^ spectral width centered at 380 and 1100 cm^–1^ was integrated, background-subtracted, and summed up for the total cellulose Raman signal. Rather than just using one characteristic Raman peak for a species (cellulose, hemicellulose, lignin, and resin), the analyses included 2 Raman peaks for cellulose, 4 for hemicellulose, 4 for lignin, and 2 for resin (Supporting Information, Table S2). To validate the Raman method development, the integrated Raman intensity of each species was compared to the wet chemistry analysis results (Figure 9), revealing a strong correlation between the two methods. This result showed strong consistency between the Raman signals and the content of the corresponding species for all the anatomic fractions. The Raman intensity was used to estimate the cellulose, hemicellulose, lignin, and resin content in the heat-treated samples (Figures 10 and 11). Using the same method, the Raman signals for cellulose, hemicellulose, lignin, and resin were extracted from spectra for the heated samples at 250 and 375 °C (Tables S3 and S4). Using the same scale in Figure 1, the cellulose, hemicellulose, lignin, and resin content in those samples were estimated based on the species’ Raman signals (Figures 10 and 11). In Figures 2 and 3, the chemical analysis results from 22 °C are also plotted to evaluate the effects of elevated temperature.

**Figure 9:**
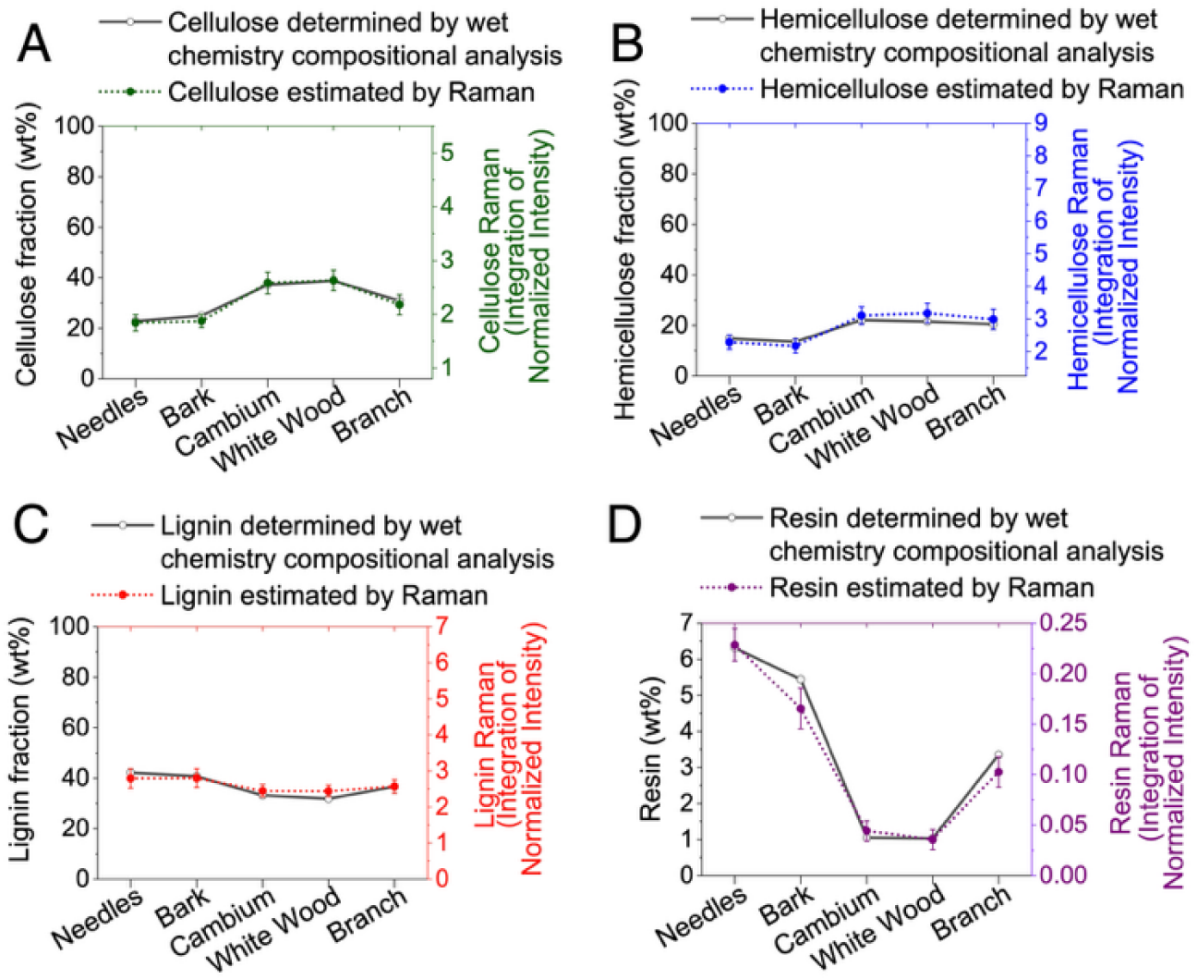
Estimation of the cellulose (A), hemicellulose (B), lignin (C), and resin (D) content in the anatomical fractions at room temperature using Raman spectroscopy. The chemical analysis results obtained at room temperature are shown for comparison.

**Figure 10:**
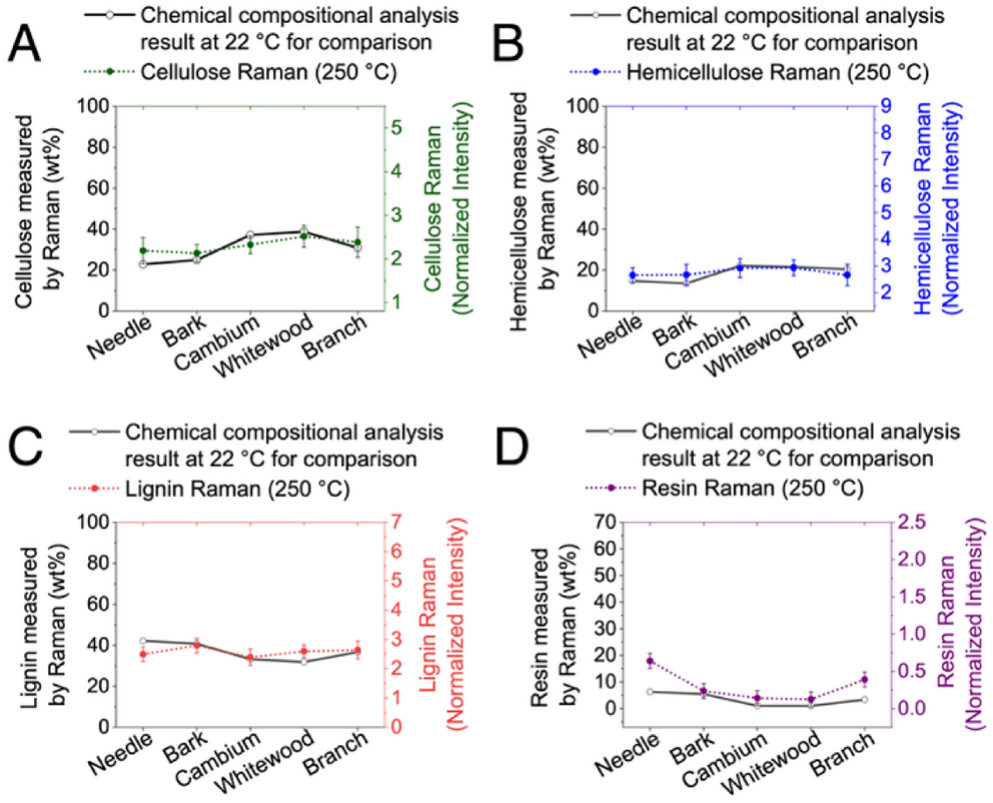
Estimation of cellulose (A), hemicellulose (B), lignin (C), and resin (D) contents in the anatomical fractions at 250 °C using Raman spectroscopy. The chemical analysis results obtained at room temperature are shown for comparison.

**Figure 11:**
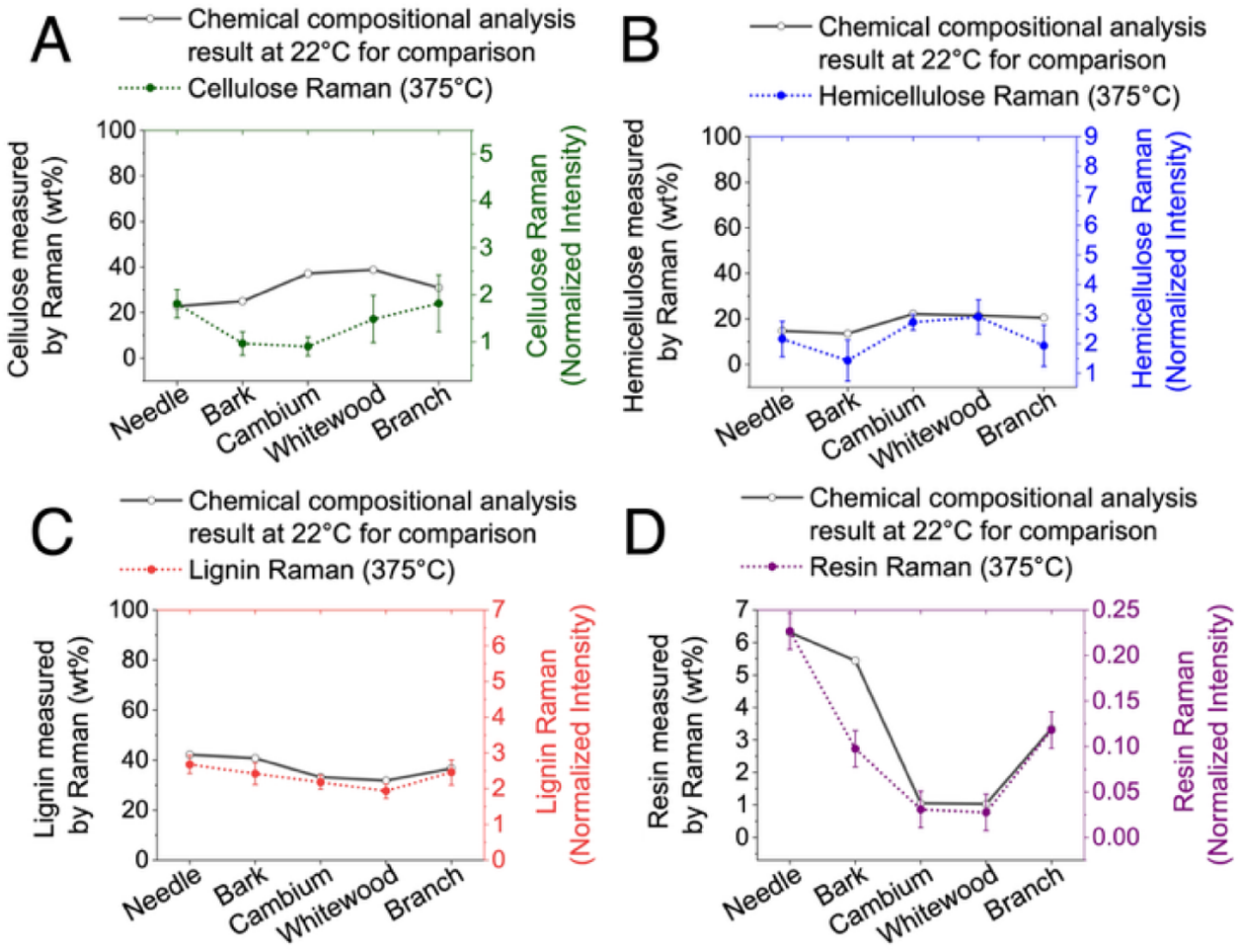
Estimation of cellulose (A), hemicellulose (B), lignin (C), and resin (D) contents in the anatomical fractions at 375 °C using Raman spectroscopy. The chemical analysis results obtained at room temperature are shown for comparison.

Figure 9 compares estimated chemical compositions derived from Raman spectroscopy at room temperature to bulk chemical analysis data. This comparative assessment validates the reliability of Raman-based estimations, demonstrating close correspondence between spectroscopic and bulk measurements for cellulose, hemicellulose, lignin, and resin content across anatomical fractions. In Figure 10, Raman-based chemical composition estimates at 250 °C highlight the initial stages of thermal degradation.

Cellulose and resin contents notably decrease, suggesting that early decomposition processes initiate at relatively moderate temperatures. These chemical changes precede significant morphological and structural alterations observed at higher temperatures. Figure 11 further elaborates on chemical transformations observed at 375 °C, revealing marked reductions in cellulose, hemicellulose, and resin acid signals, indicative of advanced thermal degradation.

These chemical shifts strongly align with extensive morphological degradation, fissuring, and surface roughening documented through microscopy, underscoring the interconnectedness of chemical and physical transformations in pyrolysis.

### 3D SRS Reveals Lignin Consolidation and Structural Alterations

Finally, Figure 12 employs stimulated Raman scattering (SRS) microscopy at 1600 cm^–1^ to visualize the lignin distribution changes upon thermal treatment. The images display 3D volume renderings of the distribution of the Raman lignin signal within pine particles.

**Figure 12:**
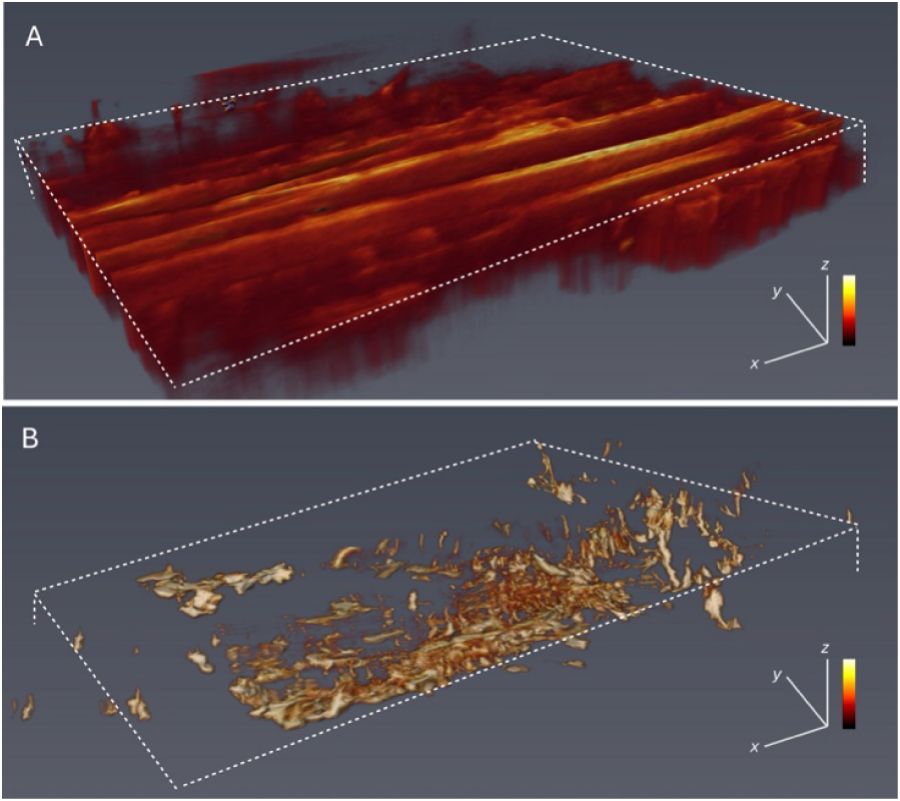
3D SRS lignin volume visualization at 1600 cm^–1^. (A) Pine particles at room temperature. (B) Pine particles after incubation at 375 °C. Dashed lines estimate the particle margins. Scale bar: 50 μm.

Comparing room temperature (Figure 12A) and 375 °C conditions (Figure 12B), the images reveal clear lignin coalescence and structural rearrangements, highlighting profound internal molecular reorganization within particles. This lignin restructuring likely contributes significantly to the overall particle integrity loss and agglomeration tendencies in thermal biomass processing systems. Collectively, these detailed morphological, textural, and chemical characterizations underscore the profound impacts of indirect heating on pine biomass, forming potential engineering solutions to mitigate feed screw plugging in biomass pyrolysis operations.

## Conclusions

Here, we present a correlative, particle-specific workflow that recreates feeder-style, indirect thermal exposure (22–500 °C) and directly links temperature-specific morphology and surface-chemistry changes in pine residues to plugging risk. By combining in situ hot-stage imaging with quench-point sampling and paired SEM stereometry and Raman spectroscopy on the same particles, we identify a potential transformation window (250–375 °C) where roughening, fissuring, and redeposited resin/bio-oil droplets emerge. These tissue-specific changes, including early changes in needles/cambium and pronounced roughness increases in whitewood, provide a mechanistic basis for increased friction and agglomeration. These results suggest that cooling or residence-time control in screw feeders to avoid these critical temperature windows can offer a practical route to reduce feed-screw plugging.

The findings presented in this study demonstrate significant morphological and chemical changes occurring in loblolly pine biomass, as it undergoes indirect heating during feed screw conveyance to pyrolysis reactors. Specifically, temperatures around 375 °C result in substantial particle shrinkage, the emergence of large fissures, and notable surface roughening across anatomical fractions, such as cambium and whitewood. Additionally, the formation of bio-oil droplets observed on particle surfaces, particularly abundant on cambium tissues, confirms premature biomass degradation, exacerbating particle agglomeration, and feed screw plugging. Raman spectroscopy further elucidated these morphological observations, highlighting cellulose, hemicellulose, and resin acid content reductions, and suggesting substantial chemical transformations at this temperature.

These observations underline the importance of temperature management within biomass feed systems. Preventing premature biomass degradation through engineered cooling systems could significantly reduce the particle agglomeration responsible for feed screw plugging. Such interventions would enhance the overall reactor efficiency and productivity, making biomass pyrolysis a more robust and economically viable renewable energy solution. Future studies should focus on optimizing feed screw designs and cooling strategies to mitigate agglomeration risks, alongside detailed analyses of bio-oil composition and its interactions with biomass particles under various thermal regimes.

## Supplementary Material



## Notes

Table S1: Raman band assignments; Table S2: Raman signal intensity at 22 °C; Table S3: Raman signal intensity at 250 °C; Table S4: Raman signal intensity at 375 °C; Figure S1: Boxplots of Sp (maximum peak height) and Sv (maximum valley depth) surface roughness values obtained from stereometric 3D reconstructions at 22 °C, 250 °C, and 375 °C; Figure S2: Bright-field optical micrograph time series of branch, needle, and bark. Scale bar = 200 μm (PDF)

## References

[1] White, E. M. Woody Biomass for Bioenergy and Biofuels in the United States – a Briefing Paper; PNW-GTR-825; U.S. Department of Agriculture: Portland, OR, 2010; p PNW-GTR-825 10.2737/PNW-GTR-825.

[2] Bahng, M. K. ; Jarvis, M. ; Haas, T. ; Nimlos, M. R. ; Donohoe, B. Studies On Heat Transfer Effects In The Pyrolysis Of Thick Wood Particles Using FT-IR Imaging; American Chemical Society: NW, WASHINGTON, 2010.

[3] Pan S., Pu Y., Foston M., Ragauskas A. J. (2013). Compositional Characterization and
Pyrolysis of Loblolly Pine and Douglas-Fir Bark. Bioenerg. Res..

[4] Mohan D., Pittman C. U., Steele P. H. (2006). Pyrolysis of Wood/Biomass
for Bio-Oil:
A Critical Review. Energy Fuels.

[5] Bridgewater V. A. (2004). Biomass
Fast Pyrolysis. Therm Sci..

[6] Haas T. J., Nimlos M. R., Donohoe B. S. (2009). Real-Time
and Post-Reaction Microscopic
Structural Analysis of Biomass Undergoing Pyrolysis. Energy Fuels.

[7] Herguido J., Corella J., Gonzalez-Saiz J. (1992). Steam Gasification
of Lignocellulosic
Residues in a Fluidized Bed at a Small Pilot Scale. Effect of the
Type of Feedstock. Ind. Eng. Chem. Res..

[8] Beaumont O., Schwob Y. (1984). Influence of Physical
and Chemical-Parameters on Wood
Pyrolysis. Ind. Eng. Chem. Proc..

[9] Bridgwater A. V. (1999). Principles
and Practice of Biomass Fast Pyrolysis Processes for Liquids. J. Anal. Appl. Pyrol..

[10] Huang F., Singh P. M., Ragauskas A. J. (2011). Characterization
of Milled Wood Lignin
(MWL) in Loblolly Pine Stem Wood, Residue, and Bark. J. Agric. Food Chem..

[11] Bonelli P. R., Della Rocca P. A., Cerrella E. G., Cukierman A. L. (2001). Effect
of Pyrolysis Temperature on Composition, Surface Properties and Thermal
Degradation Rates of Brazil Nut Shells. Bioresour.
Technol..

[12] Saha N., Klinger J., Rowland S. M., Dunning T., Carpenter D., Mills Z., Parks J. (2023). Influence of Feedstock
Variability
on Thermal Decomposition of Forest Residue in a Screw Feeder for High
Temperature Conversion. Fuel Process. Technol..

[13] Gao W., Zhang M., Wu H. (2018). Bed Agglomeration
during Bio-Oil
Fast Pyrolysis in a Fluidized-Bed Reactor. Energy
Fuels.

[14] Wang R., He X., Lin L., Flores-Betancourt A., Lee K., Dunning T., Qu J. (2023). Investigation
of Biomass Fouling on Screw Feeder in Preconversion
of Pyrolysis. ACS Sustainable Chem. Eng..

[15] Wang P., Howard B. H. (2018). Impact of Thermal Pretreatment Temperatures on Woody
Biomass Chemical Composition, Physical Properties and Microstructure. Energies.

[16] Wu R., Beutler J., Price C., Baxter L. L. (2020). Biomass Char Particle
Surface Area and Porosity Dynamics during Gasification. Fuel.

[17] Fu, P. ; Hu, S. ; Xinag, J. ; Sun, L. ; Yang, T. ; Zhang, A. ; Wang, Y. ; Chen, G. Effects of Pyrolysis Temperature on Characteristics of Porosity in Biomass Chars. In 2009 International Conference on Energy and Environment Technology; IEEE: Guilin, China, 2009; Vol. 1, pp 109–112 10.1109/ICEET.2009.33.

[18] Beaumont O., Schwob Y. (1984). Influence of Physical and Chemical Parameters on Wood
Pyrolysis. Ind. Eng. Chem. Proc. Des. Dev..

[19] Oasmaa A., Solantausta Y., Arpiainen V., Kuoppala E., Sipilä K. (2010). Fast Pyrolysis
Bio-Oils from Wood and Agricultural Residues. Energy Fuels.

[20] Oasmaa A., Kuoppala E., Gust S., Solantausta Y. (2003). Fast Pyrolysis
of Forestry Residue. 1. Effect of Extractives on Phase Separation
of Pyrolysis Liquids. Energy Fuels.

[21] Thangalazhy-Gopakumar S., Adhikari S., Ravindran H., Gupta R. B., Fasina O., Tu M., Fernando S. D. (2010). Physiochemical Properties of Bio-Oil Produced at Various
Temperatures from Pine Wood Using an Auger Reactor. Bioresour. Technol..

[22] Oasmaa A., Fonts I., Pelaez-Samaniego M. R., Garcia-Perez M. E., Garcia-Perez M. (2016). Pyrolysis Oil Multiphase Behavior
and Phase Stability:
A Review. Energy Fuels.

[23] Schindelin J., Arganda-Carreras I., Frise E., Kaynig V., Longair M., Pietzsch T., Preibisch S., Rueden C., Saalfeld S., Schmid B., Tinevez J. Y., White D. J., Hartenstein V., Eliceiri K., Tomancak P., Cardona A. (2012). Fiji: An Open-Source
Platform for Biological-Image Analysis. Nat.
Methods.

[24] Lowe D. G. (2004). Distinctive
Image Features from Scale-Invariant Keypoints. Int. J. Comput. Vis..

[25] Murtin C., Frindel C., Rousseau D., Ito K. (2018). Image Processing for
Precise Three-Dimensional Registration and Stitching of Thick High-Resolution
Laser-Scanning Microscopy Image Stacks. Comput.
Biol. Med..

[26] R Development Core Team. R. R: A Language and Environment for Statistical Computing; R Development Core Team, 2019

[27] Gomez-Rubio V. (2018). Generalized
Additive Models: An Introduction with R. J.
Stat. Software.

[28] Chinga G., Johnsen P. O., Dougherty R., Berli E. L., Walter J. (2007). Quantification
of the 3D Microstructure of SC Surfaces. J.
Microsc..

[29] Bennis H., Benslimane R., Vicini S., Mairani A., Princi E. (2010). Fibre Width
Measurement and Quantification of Filler Size Distribution in Paper-Based
Materials by SEM and Image Analysis. J. Electron
Microsc..

[30] Carpenter D., Westover T. L., Czernik S., Jablonski W. (2014). Biomass Feedstocks
for Renewable Fuel Production: A Review of the Impacts of Feedstock
and Pretreatment on the Yield and Product Distribution of Fast Pyrolysis
Bio-Oils and Vapors. Green Chem..

[31] Sluiter, J. ; Sluiter, A. D. Summative mass closure: laboratory analytical procedure (LAP) review and integration; National Renewable Energy Laboratory: Golden, 2011.

