# Supplementary material for: Surface Chemistry and Particle Morphology Changes in Pine Biomass under Indirect Thermal Gradients: Implications for Feed Screw Design
Source: ACS Omega. 2025 Dec 2;10(49):60697–706. doi: 10.1021/acsomega.5c08658 (PMC12713422; doi:10.1021/acsomega.5c08658)
Supplement: Supplementary file 1 [file ao5c08658_si_001.pdf]

Surface Chemistry and Particle Morphology Changes of Pine Biomass Under Indirect Thermal Gradients: Implications for Feed Screw Design

Yining Zeng[1], Josephine Gruber[2], Tim Dunning[1], Steven Rowland[1], Daniel Carpenter[1], and Bryon S. Donohoe[2]**

1–Renewable Resources and Enabling Sciences Center, National Renewable Energy Laboratory Golden, CO 80401, USA.

2–Biosciences Center, National Renewable Energy Laboratory, Golden, CO 80401, USA.

*To whom correspondence may be addressed: Yining.Zeng@nrel.gov and Bryon.Donohoe@nrel.gov.

Supporting Information Summary

Figure S1	pg S2
Figure S2	pg S3
Table S1	pg S4
Table S2	pg S4
Table S3	pg S5
Table S4	pg S5

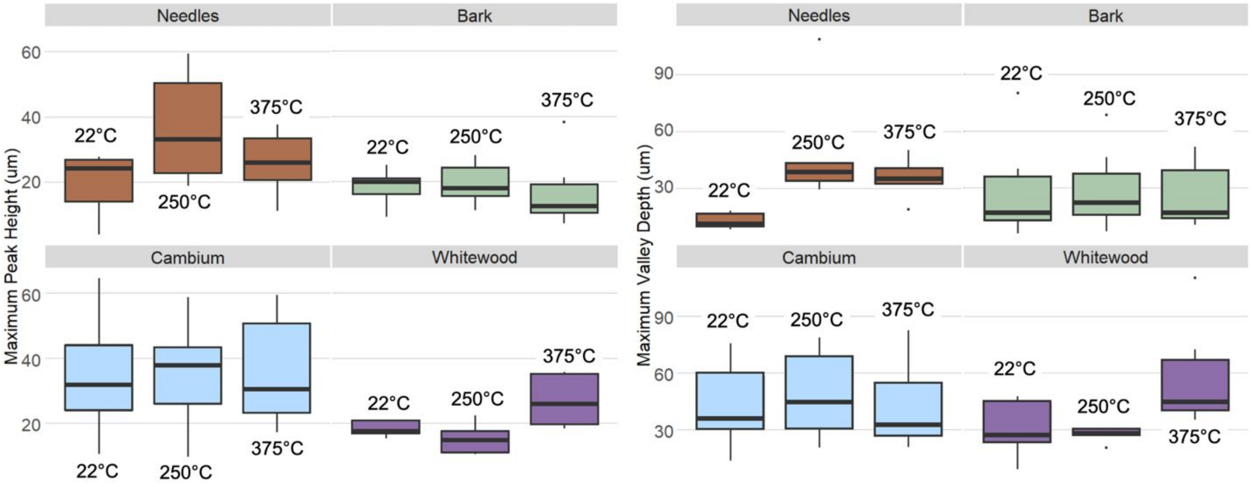

Figure S1. Boxplots of Sp (maximum peak height) and Sv (maximum valley depth) surface roughness values obtained from stereometric 3D reconstructions at 22ºC, 250ºC, and 375ºC.

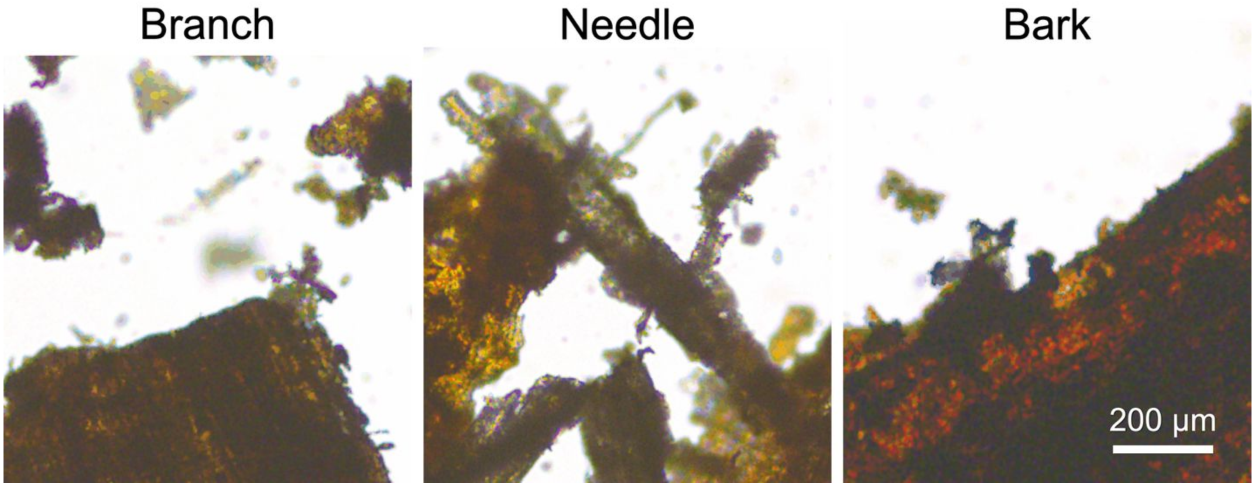

Figure S2. Bright field optical micrograph time series of branch, needle, and bark. Scale bar = 200 µm.

Table S1. Raman band assignments and locations where the background was determined.

Species / Raman Shift (cm^{-1})	Cellulose		Hemicellulose				Lignin				Resin	
Center peak location	380	1100	920	1007	1219	1471	1600	1650	1380	1432	1660	1643
Background subtraction location	400	1050	970	970	1200	1530	1620	1620	1450	1450	1620	1620

Table S2. Total Raman signal intensity calculation for cellulose, hemicellulose, lignin and resin at 22ºC.

Sample	Cellulose			Hemicellulose					Lignin					Resin		
	Total Cellulose Intensity	380 cm^{-1} Intensity	1100 cm^{-1} Intensity	Total Hemicellulose Intensity	920 cm^{-1} Intensity	1007 cm^{-1} Intensity	1219 cm^{-1} Intensity	1471 cm^{-1} Intensity	Total lignin Intensity	1600 cm^{-1} Intensity	1650 cm^{-1} Intensity	1380 cm^{-1} Intensity	1432 cm^{-1} Intensity	Total resin Intensity	1660 cm^{-1} Intensity	1643 cm^{-1} Intensity
Needle	1.84244	0.79244	1.05	2.28684	0.74948	0.9006	0.52812	0.10864	2.79504	0.94208	0.17732	1.27024	0.4054	0.22856	0.10426	0.1243
Bark	1.87336	0.70076	1.1726	2.168	0.79184	0.83408	0.47984	0.06224	2.80324	0.958	0.1884	1.26312	0.39372	0.1654	0.08219	0.08321
Cambium	2.58256	1.30388	1.27868	3.10232	1.2382	0.81708	0.58304	0.464	2.44744	0.9494	0.1764	1.08204	0.2396	0.0442	0.02266	0.02154
Whitewood	2.63088	1.34868	1.2822	3.17608	1.15284	0.86224	0.68368	0.47732	2.44148	0.9486	0.1736	1.08492	0.23436	0.03564	0.01996	0.01568
Branch	2.17848	1.06348	1.115	2.98856	1.3932	0.654	0.5946	0.34676	2.57072	0.98548	0.14884	1.07888	0.35752	0.1026	0.0514	0.0512

Table S3. Total Raman signal intensity calculation for cellulose, hemicellulose, lignin and resin for anatomical fractions at 250°C.

Sample	Cellulose			Hemicellulose					Lignin					Resin		
	Total Cellulose Intensity	380 cm^{-1} Intensity	1100 cm^{-1} Intensity	Total Hemicellulose Intensity	920 cm^{-1} Intensity	1007 cm^{-1} Intensity	1219 cm^{-1} Intensity	1471 cm^{-1} Intensity	Total lignin Intensity	1600 cm^{-1} Intensity	1650 cm^{-1} Intensity	1380 cm^{-1} Intensity	1432 cm^{-1} Intensity	Total resin Intensity	1660 cm^{-1} Intensity	1643 cm^{-1} Intensity
Needle	2.18972	1.03856	1.15116	2.65612	0.87768	1.0186	0.5868	0.17304	2.494	0.84284	0.5534	0.64324	0.45452	0.63916	0.31456	0.3246
Bark	2.13064	0.88732	1.24332	2.667	0.82764	1.09608	0.54284	0.20044	2.78772	0.56564	0.3872	1.5308	0.30408	0.23568	0.11008	0.1256
Cambium	2.32616	1.07392	1.25224	2.91716	1.0728	0.95012	0.59904	0.2952	2.38804	0.67808	0.33672	1.07716	0.29608	0.14055	0.12162	0.01893
Whitewood	2.51564	1.15528	1.36036	2.92705	1.14241	0.96096	0.59928	0.2244	2.59368	0.76128	0.27848	1.26232	0.2916	0.12412	0.11088	0.01324
Branch	2.38356	1.04568	1.33788	2.65892	1.28164	0.96944	0.17676	0.23108	2.6346	0.671	0.50336	1.03776	0.42248	0.39056	0.20096	0.1896

Table S4. Total Raman signal intensity calculation for cellulose, hemicellulose, lignin and resin for anatomical fractions at 375°C.

Sample	Cellulose			Hemicellulose					Lignin					Resin		
	Total Cellulose Intensity	380 cm^{-1} Intensity	1100 cm^{-1} Intensity	Total Hemicellulose Intensity	920 cm^{-1} Intensity	1007 cm^{-1} Intensity	1219 cm^{-1} Intensity	1471 cm^{-1} Intensity	Total lignin Intensity	1600 cm^{-1} Intensity	1650 cm^{-1} Intensity	1380 cm^{-1} Intensity	1432 cm^{-1} Intensity	Total resin Intensity	1660 cm^{-1} Intensity	1643 cm^{-1} Intensity
Needle	1.81298	0.82334	0.98964	2.16152	0.77568	0.84008	0.43736	0.1084	2.67896	0.5502	0.5234	1.19552	0.40984	0.22676	0.12136	0.1054
Bark	0.9674	0.39832	0.56908	1.43376	0.27052	0.31064	0.456	0.3966	2.42208	1.60028	0.16392	0.44644	0.21144	0.09748	0.04917	0.04831
Cambium	0.9032	0.38752	0.51568	2.72716	0.87776	0.84148	0.46804	0.53988	2.18216	0.8008	0.2096	0.89388	0.27788	0.0308	0.01526	0.01554
Whitewood	1.48504	0.48348	1.00156	2.90716	1.03748	0.87124	0.91192	0.08652	1.9332	1.14616	0.08628	0.43688	0.26388	0.02752	0.01179	0.01573
Branch	1.82064	0.65628	1.16436	1.93539	0.97716	0.41099	0.4392	0.10804	2.45956	0.90016	0.0702	1.08644	0.40276	0.118	0.0599	0.0581
